# Difficult to control epilepsy in Young Female: a common problem in a low resource setting

**DOI:** 10.11604/pamj.2014.18.212.4818

**Published:** 2014-07-08

**Authors:** Innocent Lule Segamwenge, Ngalyuka Paul Kioko

**Affiliations:** 1 Department of Internal Medicine, Intermediate Hospital Oshakati, Private Bag 5501, Oshakati, Namibia

**Keywords:** Epilepsy, seizures, neurocysticercosis

## Image in medicine

13-year old girl presented to the medical outpatient department with epilepsy diagnosed within the preceding 3 months. However, her seizures were becoming more frequent despite being on Carbamazepine and Phenobarbital at maximal tolerable dosages. She had no recent history of head trauma neither any other medical illness. Her physical examination was unremarkable. Her full blood count, liver and kidney function tests were within normal limits. A Brain CT scan was ordered which revealed multiple calcified and vesicular cysts within the Brain parenchyma; features consistent with Neurocysticercosis. The stool examination was negative for Taenia species ova. She was treated with Albendazole 400mg tid for 1 month and subsequent CT scans at 4 weekly intervals showed marked reduction in the number of active (vesicular cysts). Brain CT scans showing multiple vesicular cysts before treatment with Albendazole (A and B) and after treatment (C and D)

**Figure 1:**
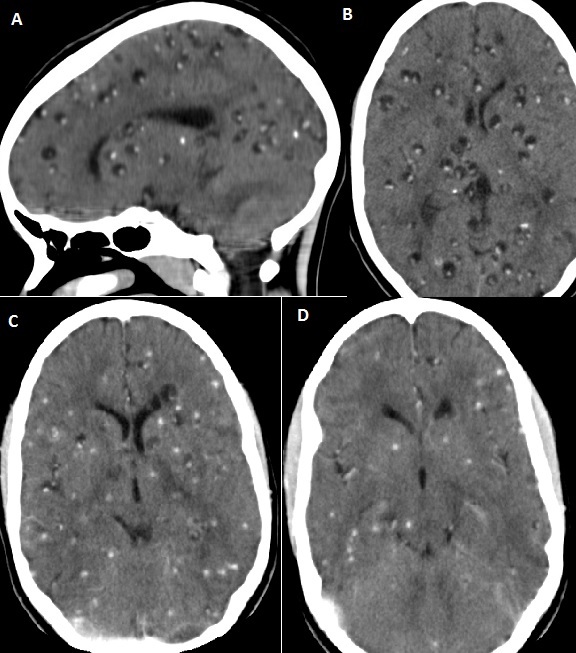
A) Brain CT(Sagittal view) showing Multiple Vesicular Cysts and Calcifications of Neurocysticercosis before treatment; B) Brain CT(axial view) showing Multiple Vesicular Cysts and Calcifications of Neurocysticercosis before treatment; C) Brain Ct (Axial View) showing resolution of almost all vesicular cysts with residual calcifications; D) Brain Ct after treatment

